# Bulletin No. 64, of the Virginia Agricultural Experiment Station

**Published:** 1897-09

**Authors:** 


### REVIEWS.

Bulletin No. 64, of the Virginia Agricultural Experiment Station.

This bulletin is prepared by Veterinarian E. P. Niles, and gives a concise account of the parasitical diseases of sheep in Virginia. As these lower forms of life cause very heavy losses by death and shrinkage in value to the sheep-owners and raisers, it is of the utmost importance to spread general information about them, so that precautionary measures may be adopted, so far as possible, to lessen the burdens to flock-owners. Veterinarians will find in many of these bulletins very useful information about these forms of diseases, and they should, as far as possible, possess them, and thus keep better posted on the nature, extent, and better methods of dealing with these scourges.

